# Telemedicine Offers Solutions for the Rural Disparities in Infectious Disease (ID) Care Delivery

**DOI:** 10.1093/ofid/ofaf052

**Published:** 2025-02-04

**Authors:** Christian Perez

**Affiliations:** Department of Medicine—Division of Infectious Diseases, University of Pittsburgh Medical Center, Pittsburgh, Pennsylvania, USA

**Keywords:** decline in ID specialists, ID fellowship match, medical education, socioeconomic disparities, telemedicine

## Abstract

In the United States, there is a growing shortage of infectious diseases (ID) physicians that highlights disparity between rural versus urban ID expertise resulting in a healthcare gap with significant consequences for patients unable to have ID directed care available to them. Telemedicine is a crucial modality of healthcare that can be used to bridge that gap by providing quality care consistent with outcomes for inpatient ID service models, while also serving as a tool for much greater geographic coverage by a single ID physician. As a specialty, we must embrace telemedicine for the good of our patients, whose local communities may not have access to ID care, and to create the incentive of broader community impact for potential entrants into the ID field.

Even before the crush of the COVID-19 pandemic, many within the infectious diseases (ID) community were sounding the alarm about the growing gap between supply and demand for ID expertise. The COVID-19 emergency further widened this gap and exposed major deficiencies in US healthcare and public health systems. Out of necessity, the pandemic forced rapid adoption of novel modalities, of which health systems have been traditionally slow to embrace. The foremost instance of rapid adoption was telemedicine, which was initially thought to be a bridging strategy until the pandemic ran its course, but it has emerged as a potential cornerstone for filling gaps in ID care. Here we review the reasons behind the gaps in ID expertise and how tele-infectious disease (tele-ID) care can help close the gaps in ID care delivery. The professional advantages for physicians of providing remote ID care rather than traditional in-person practice have recently been highlighted and will not be covered [1].

## THE PROBLEM: TOO FEW ID PHYSICIANS

National attention to the shortage of ID physicians can be traced back to 2014–2015, when less than half of all fellowship programs filled their training slots [2]. In 2015, the Infectious Diseases Society of America along with George Washington University Health Workforce Institute conducted a survey of ID fellows completing their training and active ID physicians to ascertain the reasons for dwindling enthusiasm for ID as a specialty. The primary reason noted (48.6%) was lack of practice opportunities in desired locations. Further analysis suggested a trend to prefer the local job market around training areas rather than smaller cities or rural communities [3]. Despite modest increases in overall matched ID fellowship applicants in the recent years since this survey, the 2024 match did not augur well for the future. Only 50.8% of programs filled their training slots, which was the worst outcome since 2014–2015. The recent match results were even worse for pediatric ID programs, with only 42.4% filling their slots [4].

If more ID physicians are not trained, it will prove difficult to address the disparity in ID care between rural and urban America through traditional in-person practice models. In 2017, 312 (9.9%) and 499 (79.5%) of all US counties had either below-average ID physician densities or no ID physicians, respectively. This equates to 208 million citizens living in counties with scarce to no ID physician coverage [5]. It is not surprising that rural counties accounted for this huge gap in ID coverage given the preferences of trainees and physicians revealed in the Infectious Diseases Society of America/George Washington University Health Workforce Institute surveys [6]. Without ID specialists in rural areas, there is well-documented higher risk of mortality, longer length of hospital stay, readmission, and higher costs for many types of infections including *Staphylococcus aureus* bacteremia [7], multidrug resistant Gram-negative infections [8], enterococcal bacteremia [9], and candidemia [10]. To reduce disparities in ID care between rural and urban America and to address the worsening shortage of ID physicians, new models of care delivery need to be embraced.

### Help From Tele-ID Care

Telemedicine is an innovative care modality that is well-suited for the ID specialty. Most inpatient ID care plans can be formulated initially through thorough review of the medical record with corroboration by verbal history and remote physical examination using advanced video technology. This approach can be rendered virtually and has been shown to have similar or better clinical efficacy as in-person consults [11, 12]. More importantly, all forms of tele-ID, whether via patient-to-physician video conferencing with onsite nurse-mediated physical examination (video consult), asynchronous chart review with documentation (e-consult), or physician-to-physician telephonic consultation (telephonic consult), have been shown to have significant impact at rural medical centers that are without local ID support [13]. For example, Tande and colleagues showed improved mortality outcomes using asynchronous ID e-consults, while also decreasing transfer rates to tertiary centers [14]. In addition, Monkowski and colleagues established decreased length of stay at rural hospitals when using real-time interactive telemedicine assessments [15]. Telemedicine has also been effective for antibiotic stewardship. Vento and colleagues explicated reductions in the use of vancomycin, meropenem, and fluoroquinolones through a combination of asynchronous (e-consult) and real-time consultation modalities, working in collaboration with a multidisciplinary stewardship team across 16 remote hospital centers [16]. Last, there is evidence that telemedicine can increase a single physician's productivity, which is needed to help bridge the gap in access to ID expertise [17]. Although these attributes of tele-ID may help reduce the gaps in access to ID care, the shortage of ID physicians still remains a critical challenge. In addition, acceptance of remote ID care by patients and other providers, compared with traditional in-person care, is likely to vary by community and can pose a barrier to care. Finally, additional studies are needed to assess the quality and effectiveness of tele-ID care across different type of providers (physicians, advanced practice providers and ID pharmacists), locations, and types of ID.

### Growth of Tele-ID Should Be Encouraged

For the millions of Americans living in rural areas of our country, tele-ID provides an opportunity for specialty care without the burden of travel to urban healthcare centers. For future graduating ID fellows and medical residents considering the specialty, tele-ID provides the opportunity to live in desired locations, practice from home, and contribute to care of all Americans regardless of where patients live. There is mounting clinical evidence supporting tele-ID as an effective alternative to traditional on-site/in-person practice, but it is up to us as ID physicians to expand its use. The future of ID care is at a critical nexus. We must expand access to ID care and improve the attractiveness of ID practice by leveraging available technology to increase its reach and appeal for providers.

## Note

***Potential conflicts of interest.*** C.P. is employed by University of Pittsburgh Physicians of UPMC and provide telemedicine ID consultation through IDConnect, Inc. He also holds positions as Associate Clinical Director and Director of Telemedicine Physician Training and Education for IDConnect, Inc. He has no financial interest in ID Connect, Inc. C.P. does not have financial conflicts to disclose or funding sources aside from University of Pittsburgh Physicians of UPMC.
